# Postoperative outcomes, predictors and trends of mortality and morbidity in patients undergoing hip fracture surgery with underlying aortic stenosis: a nationwide inpatient sample analysis

**DOI:** 10.1186/s12872-023-03584-2

**Published:** 2023-11-03

**Authors:** Shahzad Hassan, Waqas Anwar, Shivani Mehta, Muhammad Iftikhar Hanif, Abdallah Kamouh, Alexander J. Blood

**Affiliations:** 1 Boston Medical Center, One Boston Medical Center Pl, Boston University School of Medicine, , Boston, MA 02118 USA; 2 Rawalpindi Medical University, Rawalpindi, 46000 Punjab Pakistan; 3 Department of Internal Medicine, Wayne State University/Trinity Health Oakland, Pontiac, MI 48341 USA; 4 Department of Interprofessional Health Sciences and Health Administration, Seton Hall University, South Orange, NJ 07079 USA; 5 Department of Medicine, Division of Cardiology, Medical University of South Carolina, Florence, SC 29505 USA; 6 Department of Medicine, Division of Cardiology, Newton Wellesley Hospital, Newton, MA 02462 USA; 7 Department of Medicine, Division of Cardiovascular Medicine, Boston, MA 02115 USA; 8 Harvard Medical School, Boston, MA 02115 USA

**Keywords:** Hip fracture, Aortic stenosis, Postoperative mortality, Hip fracture surgery

## Abstract

**Background:**

Hip fractures frequently necessitate hospitalization, especially among patients aged 75 and above who might concurrently suffer from aortic stenosis (AS). This study focuses on postoperative outcomes, potential determinants of morbidity and mortality, as well as evolving trends in patients with AS undergoing surgical repair of hip fractures.

**Methods:**

A retrospective analysis of the Nationwide Inpatient Sample from 2008 to 2019 was conducted. Hip fracture cases were identified, and a subgroup with AS was isolated using the ICD-9 and ICD-10 diagnostic codes. We compared baseline characteristics, postoperative in-hospital outcomes and trends in mortality and morbidity between patients with and without AS.

**Results:**

From the dataset, 2,834,919 patients with hip fracture were identified on weighted analysis. Of these, 94,270 (3.3%) were found to have concurrent AS. The AS cohort was characterized by higher mean age and elevated burden of cardiovascular comorbidities, such as coronary artery disease, peripheral vascular disease, pulmonary hypertension, congestive heart failure and cardiac arrhythmias. Postoperative mortality following hip fracture surgery was greater in the AS groups compared to non-AS group (3.3% vs 1.57%, *p* < 0.001). Risk factors such as congestive heart failure (OR, 2.3[CI, 2.1–2.6]), age above 85 years (OR, 3.2[CI, 2.2–4.7]), cardiac arrhythmias (OR, 2.4[CI, 2.2–2.6]), end-stage renal disease (OR, 3.4[CI, 2.7–4.1]), malnutrition (OR, 2.3[CI, 2.1–2.7]) and AS (OR, 1.2[CI, 1.08–1.5] were associated with increased adjusted odds of postoperative mortality. AS was linked to higher adjusted odds of postoperative mortality (OR, 1.2 [CI, 1.1–1.5]) and complications such as acute myocardial infarction (OR, 1.2 [CI, 1.01–1.4]), cardiogenic shock (OR, 2.0[CI, 1.4–2.9]) and acute renal failure (OR, 1.1[CI, 1.02–1.2]). While hospital stay duration was comparable in both groups (average 5 days), the AS group incurred higher costs (mean $50,673 vs $44,607). The presence of acute heart failure in patients with AS and hip fracture significantly increased mortality, hospital stay, and cost. A notable decline in postoperative in-hospital mortality was observed in both groups from 2008–2019 though the rate of major in-hospital complications rose.

**Conclusion:**

AS significantly influences postoperative in-hospital mortality and complication rates in hip fracture patients. While a reduction in postoperative mortality was observed in both AS and non-AS cohorts, the incidence of major in-hospital complications increased across both groups.

**Supplementary Information:**

The online version contains supplementary material available at 10.1186/s12872-023-03584-2.

## Introduction/Background

Hip fractures, representing a significant orthopedic emergency, are a common cause of hospitalization. With an annual incidence of over 340,000 fractures [1], the United States (US) has one of the highest hip fracture rates in the world [2]. The incidence of hip fractures is estimated to double worldwide by 2025 [3], mainly affecting the elderly population, particularly those aged 75 years and above [1, 4–7]. This patient population is also burdened with an elevated prevalence of cardiovascular comorbidities [8], of which aortic stenosis (AS) is the predominant valvular heart disease [9, 10].

Surgery is the principal treatment approach for hip fractures [11]. However, the presence of concurrent AS can increase the risk of perioperative major cardiovascular events and mortality [12–14]. Existing literature on the outcomes of patients with AS undergoing hip fracture surgery has produced inconsistent findings. In a retrospective study by Adunsky et al [15] on elderly patients with mean age of 86 years, AS was associated with high in-hospital postoperative mortality and major complications. Keswani et al [16] reported increased risk of perioperative complications and mortality in patients ≥ 65 years of age with moderate to severe AS undergoing hip fracture surgical treatment. Similar results are published by Rostagno et al in patients with severe AS [17]. Conversely, another case–control study did not manifest significant differences in 30-days mortality (6.2% vs 6.8%) between severe AS group and controls [18]. These single center studies are performed on elderly population with moderate to severe AS only, underscoring the need for further investigation into the association of AS with hip fracture postoperative outcomes and mortality on nationally representative data, as it can inform the guidelines for improved outcomes for this patient population.

The objective of this study was to analyze the postoperative outcomes, predictors and trends of in-hospital mortality in patients undergoing hip fracture surgery who had concomitant AS, using data from the Nationwide Inpatient Sample (NIS).

## Methods

### Study sample and design

We conducted a retrospective cohort study using the NIS database from 2008–2019. It is the largest publicly available all-payer inpatient healthcare database that is designed to produce US regional and national estimates of inpatient utilization, access, cost, quality and outcomes [19]. Unweighted, it contains data from more than 7 million hospital stays annually. Weighted, it estimates more than 35 million hospitalizations nationally. As data are deidentified and publicly available, this study is exempted from approval by the institutional review board.

**Fig. 1:**
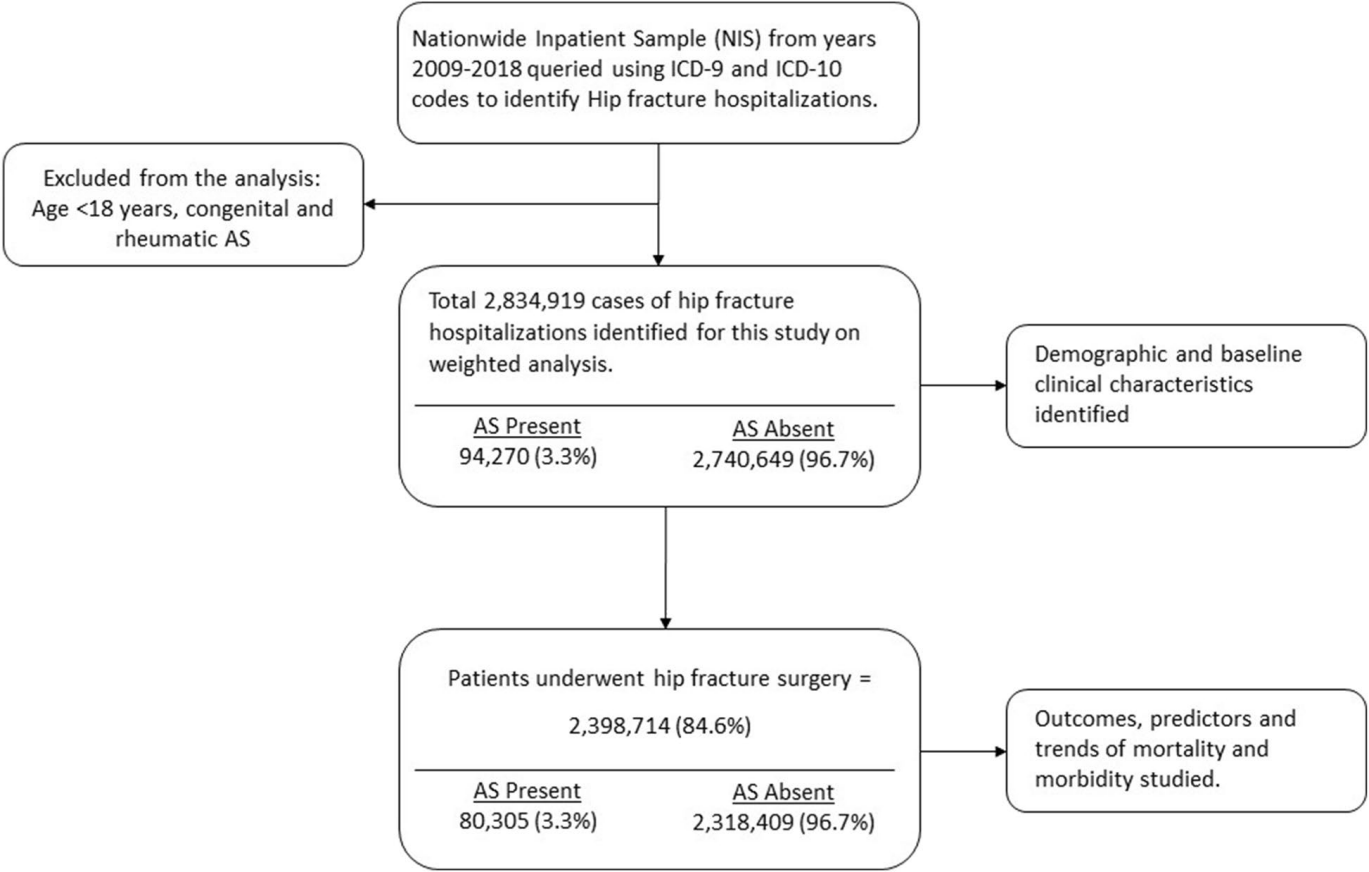
Reported numbers based on weighted analysis

Hip fracture hospitalizations were identified in the database using International Classification for disease (ICD) 9 and 10 diagnostic codes. Within the hip fracture group, a subgroup of AS was created using ICD codes (424.1, I35.0). We excluded cases with rheumatic and congenital AS (Fig. 1). Patients under the age of 18 years were also excluded from this study. Baseline demographic characteristics, including age, sex and race, are available in the dataset. Comorbidities were identified from the database using ICD 9 and 10 diagnostic codes and Charlson comorbidities as reported in the HCUP dataset (Supplementary table 1).

Within the hip fracture patient population, a subgroup of clinically significant AS was created by identifying patients who had both AS and acute or AS and acute on chronic congestive heart failure (CHF) within the same hospitalization using ICD-9 and ICD-10 diagnostic codes.

We adhered to Nationwide Database Data Use Agreement (DUA) while conducting this analysis.

### Study outcomes

The primary outcome was inpatient all-cause mortality. The secondary outcomes included acute myocardial infarction (AMI), acute pulmonary edema, cardiogenic shock, acute deep venous thrombosis/pulmonary embolism (DVT/PE), septic shock, pneumonia, acute respiratory failure, acute ischemic & hemorrhagic cerebrovascular accident (CVA), acute renal failure, acute delirium, postoperative surgical site infection (SSI) and postoperative bleeding.

### Statistical analysis

Continuous variables were reported as the mean with standard deviation (SD) or median with interquartile range (IQR). Linear regression was used to compare weighted estimates of continuous variables. Categorical variables were reported as proportions and compared using Pearson’s chi-square test.

A multivariable logistic regression model was developed to determine independent predictors of inpatient mortality in all hip fracture patients while adjusting for demographics (age, sex, race) and comorbidities: hypertension, diabetes mellitus (DM), smoking, coronary artery disease (CAD), CHF, cardiac arrhythmias, long-term anticoagulation use, end-stage renal disease (ESRD), AS, malnutrition, alcohol use disorder, chronic obstructive pulmonary disease (COPD), dementia, vitamin D deficiency and osteoporosis.

Another multivariable logistic regression model was developed to determine the association of AS with postoperative outcomes while adjusting for age, sex, race and the following comorbid conditions: pulmonary hypertension, ESRD, chronic kidney disease (CKD), dementia, CAD, smoking status, obesity, AS, osteoporosis, DM, hypertension, COPD, cardiac arrhythmias, long-term use of anticoagulation, vitamin D deficiency, alcoholism, and malnutrition.

Odds ratios (ORs) and 95% confidence intervals (CIs) were reported. Yearly trends of postoperative mortality and morbidity were studied and reported as percentages. Temporal trends were demonstrated graphically, linear trends were calculated using the Cochrane Armitage method, and p-trends were reported. Survey analysis methodology using weights of hospital-level discharge from NIS was used to calculate nationally representative estimates.

Inflation-adjusted total hospital cost was calculated from total charges and cost-to-charge files available from the healthcare cost and utilization project (HCUP) website.

A *P* value < 0.05 was considered statistically significant. We used Stata v. 17.0 (StataCorp, Longview, TX) for all statistical analyses.

## Results

### Demographics and baseline characteristics

Over the study period of 2008–2019, a total of 2,834,919 (mean (SD) age 77.3 (12.9), 68% females, 85.6% whites) patients with a primary diagnosis of hip fracture were hospitalized in the US. Of these, 94,270 (3.3%) had a concomitant diagnosis of AS. A total of 84.6% of hospitalized patients with hip fracture underwent surgery. Of the patients who underwent surgery for hip fracture, 80,305 (3.3%) had a concomitant diagnosis of AS.

Patients who had hip fracture and AS were more likely to be older (62% being 85 years or older) and female (68%) and had a higher prevalence of comorbidities such as DM, hypertension, CAD, cardiac arrhythmias, long-term anticoagulation use, pulmonary hypertension, CKD, ESRD and dementia. A total of 87.2% of hip fracture hospitalizations were noted in large urban hospitals (Table 1).

**Table 1:** Baseline characteristics of hip fracture hospitalizations

Patients, Number (%)				
		**Aortic stenosis**		***P* value**
		**Present**	**Absent**	
	2,834,919	94,270 (3.3)	2,740,649 (96.7)	
Characteristics				
Age, mean (SD), y		84.5 (6.8)	77.1 (13.0)	< 0.001
Age groups, y				< 0.001
18–54	164,800 (5.81)	265(0.28)	164,535 (6.0)	
55—64	259,385(9.1)	1,445(1.53)	257,940(9.41)	
65—74	493,485(17.4)	6,845(7.26)	486,640(17.8)	
75—84	859,835(30.3)	26,495(28.1)	833,340 (30.4)	
≥ 85	1,057,415(37.3)	59,220(62.8)	998,195(36.4)	
Sex				0.140
Female	1,834,694(67.5)	64,035 (67.9)	1,847,619 (67.4)	
Male	922,970(32.6)	30,215(32.1)	892,755(32.6)	
Race				< 0.001
White	2,320,599(85.4)	80,420(88.7)	2,240,179(85.2)	
Black	130,065(4.78)	2,510(2.77)	127,555(4.85)	
Hispanics	152,965(5.63)	4,215(4.65)	148,750 (5.66)	
Other	114,890(4.23)	3,510(3.87)	111,380(4.24)	
Comorbidities				
Hypertension	583,900 (20.6)	31,585 (33.5)	552,315 (20.2)	< 0.001
Diabetes	644,890(22.7)	23,075(24.5)	621,815(22.7)	< 0.001
Pulmonary Hypertension	125,075(4.41)	12,570(13.3)	112,505(4.1)	< 0.001
Coronary artery disease	691,380(24.4)	37,710(40.0)	653,670(24.0)	< 0.001
Peripheral vascular disease	225,220(7.94)	12,020(12.8)	213,200(7.78)	< 0.001
Congestive heart failure	478,105(16.9)	33,625(35.7)	444,480(16.2)	< 0.001
Cardiac Arrhythmias	845,705(29.8)	44,005(46.7)	801,700(29.3)	< 0.001
Long term anticoagulation	280,795(9.90)	12,400(13.2)	268,395(9.79)	< 0.001
Chronic kidney disease	460,885(16.3)	23,610(25)	437,275(16)	< 0.001
End stage renal disease	57,380(2.02)	2,435(2.58)	54,945(2.0)	< 0.001
Malignancy	32,895(3.07)	1,115(3.5)	31,780(3.06)	0.05
Obesity	148,020(5.22)	4,535(4.81)	143,485(5.24)	0.01
Alcohol Abuse	130,335(4.60)	1,830(1.94)	128,505(4.69)	< 0.001
Ataxia	95,055(3.35)	3,180(3.37)	91,875(3.35)	0.87
Osteoporosis	482,655(17)	18,100(19.2)	464,555(17)	< 0.001
Vitamin D deficiency	111,915(3.95)	3,540(3.76)	108,375(3.95)	0.16
Protein energy malnutrition	161,670(5.7)	5,610(5.95)	156,060(5.69)	0.141
Dementia	615,970(21.7)	25,600(27.2)	590,370(21.5)	< 0.001
Osteoarthritis	530,775(18.7)	20,385(21.6)	510,390(18.6)	< 0.001
Rheumatoid arthritis	76,035(2.68)	2,475(2.63)	73,560(2.68)	0.622
Smoking	160,870(5.67)	2,870(3.04)	158,000(5.77)	< 0.001
Chronic obstructive pulmonary disease	236,805(22.1)	7,045(22.1)	229,760(22.1)	0.984
Elixhauser groups^a^				< 0.001
< 4	2,176,239(76.8)	59,720(63.3)	2,116,519(77.3)	
4—6	540,020(19)	24,315(25.8)	515,705(18.8)	
> 6	118,660(4.19)	10,235(10.9)	108,425(3.96)	
Insurance status				< 0.001
Medicare	2,313,664 (81.6)	87,135 (92.4)	2,226,529(81.2)	
Medicaid	106,750(3.77)	960(1.02)	105,790(3.86)	
Private	296,215(10.4)	4,665(4.95)	291,550(10.6)	
Others	118,290(4.17)	1,510(1.6)	116,780(4.26)	
Hospital bed size^b^				0.43
Small	546,569 (19.3)	17,815(18.9)	528,754(19.3)	
Medium	851,745 (30)	28,360(30.1)	823,385(30.0)	
Large	1,436,605 (50.7)	48,095(51)	1,388,510(50.7)	
Hospital location^b^				< 0.001
Rural	364,120(12.8)	10,475(11.1)	353,645(12.9)	
Urban	2,470,800(87.2)	83,795(88.9)	2,387,005 (87.1)	

^a^Elixhauser Comorbidity Index is a method of categorizing comorbidities of patients based on ICD diagnosis codes found in administrative data. Group < 4 represents patients with fewer than 4 comorbidities, Groups 4–6 includes patients with four to six comorbidities and > 6 comprises patients with more than six comorbidities [20]

^b^Bed size categories are based on hospital beds and location is rural vs urban as defined in the NIS description of data elements

### Hip fracture surgery postoperative outcomes in patients with underlying AS

In patients undergoing hip fracture surgery who had underlying AS, 2,665 (3.3%) deaths occurred compared to 36,330 (1.57%) deaths in patients without underlying AS.

Patients with AS had more in-hospital complications, including AMI (3.97% vs 1.57%, *p* < 0.001), acute pulmonary edema (0.33% vs 0.16%, *p* < 0.001), cardiogenic shock (0.60% vs 0.16%, *p* < 0.001), pneumonia (7.5% vs 5.6% *p* < 0.001), acute respiratory failure (8.8% vs 6.0% *p* < 0.001), acute ischemic CVA (5.1% vs 2.71% *p* < 0.001) and acute renal failure (18.8% vs 12.6 *p* < 0.001) (Table 2).

**Table 2:** Outcomes post hip fracture surgery by aortic stenosis status

Outcomes	No. (%)	Aortic Stenosis		*P* value
		**Present**	**Absent**	
	2,398,714 (84.6)	80,305 (3.35)	2,318,409 (96.7)	
Length of stay—days Median (IQR)		5.0(4–7)	5.0(3–7)	< 0.001
Cost—$ Median (IQR)		50,673 (30,249–106,194)	44,607 (26,657–83,804)	0.042
In hospital Deaths	38,995(1.63)	2,665(3.3)	36,330(1.57)	< 0.001
Acute Myocardial Infarction	39,495(1.64)	3,200(3.97)	36,295(1.57)	< 0.001
Acute Pulmonary edema	3,980 (0.165)	265(0.33)	3,715(0.16)	< 0.001
Cardiogenic shock	4,330 (0.18)	490(0.60)	3,840(0.16)	< 0.001
Acute DVT/Pulmonary embolism	25,890 (1.08)	905(1.13)	24,985(1.08)	0.55
Septic shock	10,155 (0.42)	360(0.45)	9,795(0.42)	0.62
Pneumonia	137,290 (5.7)	6,025(7.5)	131,265(5.66)	< 0.001
Acute Respiratory failure	146,620 (6.1)	7,130(8.88)	139,490(6.02)	< 0.001
Acute Ischemic CVA	66,960(2.79)	4,155(5.17)	62,805(2.71)	< 0.001
Acute Hemorrhagic CVA	3,645 (0.15)	160(0.20)	3,485(0.15)	0.11
Acute Renal failure	306,800 (12.8)	15,115(18.8)	291,685(12.6)	< 0.001
Acute Delirium	64,065 (2.67)	2,890(3.6)	61,175(2.64)	< 0.001
Post operative SS infection	4,135 (0.172)	160(0.19)	3,975(0.17)	0.40
Post operative bleeding	14,495 (0.60)	790(0.97)	13,n(0.59)	< 0.001

*Abbreviations*: *IQR* Interquartile Range, *DVT* Deep Venous Thrombosis, *CVA* Cerebrovascular Accident, *SS* Surgical Site

There was no statistically significant difference in the length of hospitalization between groups.

In a multivariable logistic regression model, AS was independently associated with mortality (aOR 1.28; 95% CI 1.08–1.51, *p* = 0.003) among patients who underwent hip fracture surgery.

**Fig. 2:**
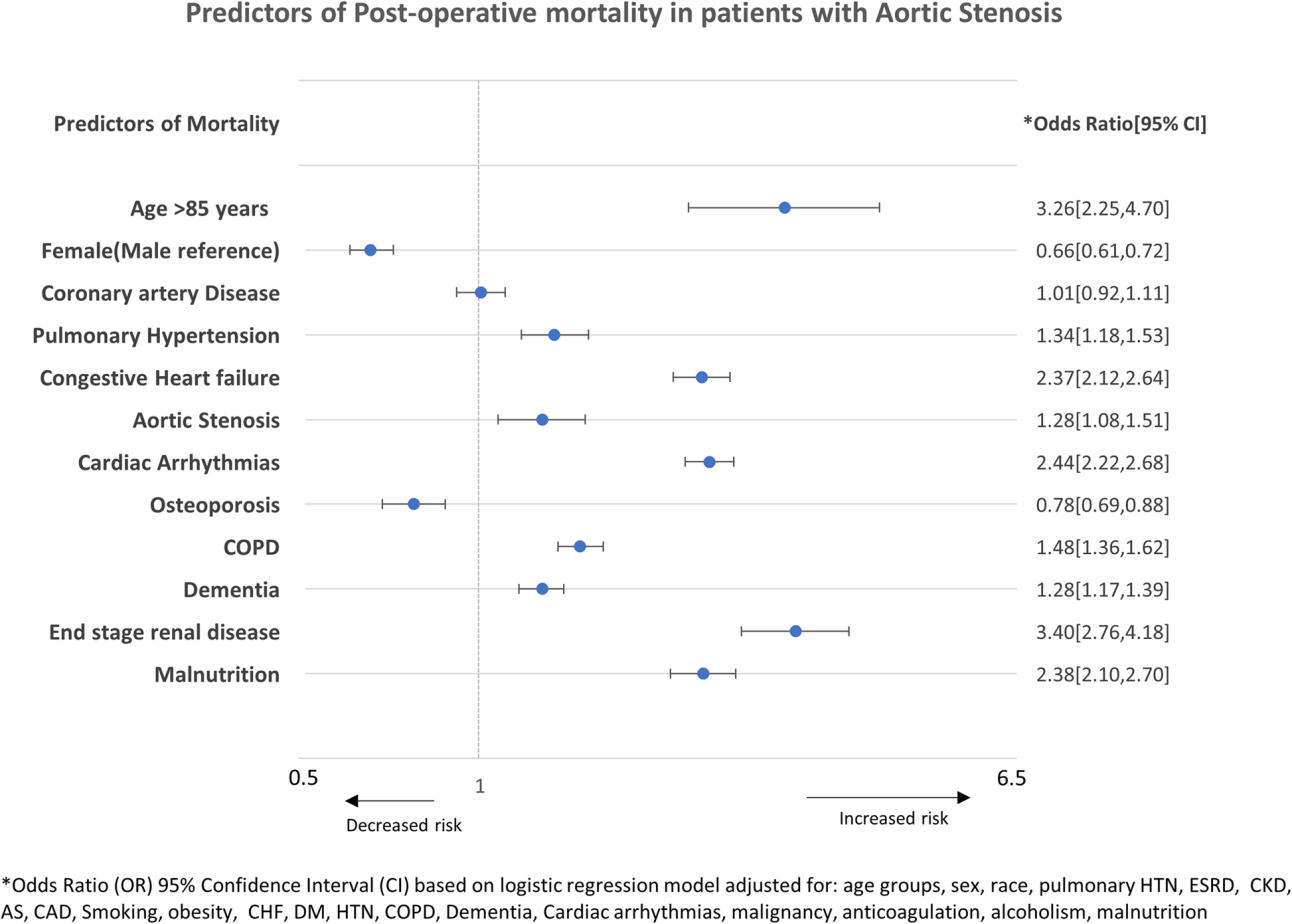
Predictors of postoperative mortality in patients with AS

Other significant predictors of mortality were age > 85 years (*p* < 0.001), pulmonary hypertension (*p* < 0.001), CKD (*p* < 0.001), ESRD (*p* < 0.001), CHF (*p* < 0.001) and malnutrition (*p* < 0.001). In the adjusted analysis, CAD was not identified as a predictor of mortality (*p* = 0.69) (Table 3, Fig. 2).

**Table 3:** Predictors of postoperative mortality in hospitalized patients with hip fracture^a^

Variables	Adjusted Odds ratio	95% Confidence Interval	*P* value
Age, years			
< 55 (Reference)			
55–64	1.32	0.87–1.99	0.183
65–74	1.70	1.16–2.46	0.006
75- 84	2.19	1.51–3.17	< 0.001
≥ 85	3.26	2.25–4.73	< 0.001
Sex			
Male (Reference)			
Female	0.66	0.61 – 0.72	< 0.001
Race			
White (Reference)			
Black	1.07	0.88–1.31	0.453
Hispanic	0.95	0.78–1.14	0.601
Others	1.02	0.84 – 1.25	0.785
Comorbidities			
Pulmonary hypertension	1.34	1.18–1.53	< 0.001
End stage renal disease	3.40	2.76–4.18	< 0.001
Chronic kidney disease	1.44	1.28–1.63	< 0.001
Coronary artery disease	1.01	0.92 – 1.11	0.691
Smoking	0.93	0.56–1.55	0.798
Obesity	0.71	0.57–0.88	0.002
Osteoporosis	0.78	0.69–0.88	< 0.001
Congestive heart failure	2.37	2.12–2.64	< 0.001
Aortic stenosis	1.28	1.08 – 1.51	0.003
Diabetes mellitus	1.01	0.92–1.11	0.725
Hypertension	0.96	0.84–1.10	0.603
Chronic obstructive pulmonary disease	1.48	1.36–1.62	< 0.001
Dementia	1.28	1.17–1.39	< 0.001
Cardiac arrhythmias	2.44	2.22–2.68	< 0.001
Malignancy	1.34	1.10–1.62	0.003
Long term anticoagulation use	0.57	0.50–0.64	< 0.001
Vitamin D deficiency	0.64	0.50–0.82	< 0.001
Alcoholism	0.69	0.45–1.04	0.078
Malnutrition	2.38	2.10–2.70	< 0.001

^a^Based on multivariable logistic regression model adjusted for demographics (age, sex, race) and comorbidities: hypertension, DM, smoking, CAD, CHF, cardiac arrhythmias, long term anti-coagulation use, ESRD, AS, malnutrition, alcohol use disorder, COPD, dementia, vitamin D deficiency and osteoporosis

In hip fracture patients with AS undergoing surgery, AS was associated with higher adjusted odds of mortality (aOR, 1.2 [CI, 1.1–1.50], *p* = 0.004), AMI (aOR, 1.2 [CI, 1.0–1.4], *p* = 0.030), cardiogenic shock (aOR, 2.0 [CI, 1.4–2.9] *p* < 0.001), and acute renal failure (aOR, 1.1 [CI, 1.0–1.19], *p* = 0.008) compared to the group undergoing hip fracture surgery without concomitant AS. In the adjusted analysis, AS was not statistically associated with acute pulmonary edema, pneumonia, septic shock, acute DVT/PE, acute postoperative bleeding, and acute delirium (Table 4).

**Table 4:** Association of AS with In-hospital postoperative outcome^a^

Outcomes	Adjusted Odds Ratio	95% Confidence Interval	*P* value
In-hospital Mortality	1.27	1.1—1.50	0.004
Acute Myocardial Infarction	1.20	1.01—1.43	0.030
Acute pulmonary edema	1.25	0.74—2.14	0.39
Pneumonia	0.95	0.85—1.07	0.48
Acute renal failure	1.10	1.02—1.19	0.008
Cardiogenic shock	2.06	1.42—2.97	< 0.001
Septic Shock	0.76	0.50—1.15	0.206
Acute DVT/PE	1.00	0.72—1.40	0.967
Acute ischemic CVA	1.04	0.78—1.40	0.737
Acute hemorrhagic CVA	1.37	0.77—2.44	0.274
Post operative bleeding	1.09	0.38—3.05	0.868
Post operative Surgical Site Infection	1.64	1.06—2.52	0.024
Acute Delirium	1.03	0.90—1.18	0.615

*Abbreviations*: *DVT* Deep venous thrombosis, *PE* Pulmonary embolism, *CVA* Cerebrovascular accident

^a^ Based on multivariable logistic regression model adjusted for age, sex, race and following comorbid conditions: pulmonary hypertension, ESRD, CKD, dementia, CAD, smoking status, obesity, AS, osteoporosis, DM, hypertension, COPD, cardiac arrhythmias, long term use of anticoagulation, vitamin D deficiency, alcoholism, and malnutrition

### Postoperative outcomes in patients with underlying clinically significant AS

In patients who underwent hip fracture surgery and had AS with acutely decompensated CHF in the same hospitalization, 485 (9.4%) deaths occurred compared to 38,510 (1.6%) among hip fracture surgery patients without AS. There was a higher prevalence of AMI (13.5% vs. 1.6%, *p* < 0.001), acute pulmonary edema (0.7% vs 0.16%, *p* < 0.001), acute respiratory failure (34% vs 6%, *p* < 0.001) and other in-hospital complications, such as septic shock, pneumonia, acute ischemic CVA, acute delirium and cardiogenic shock, compared to the group undergoing hip fracture surgery without underlying clinically significant AS. Additionally, the length of hospitalization was higher in the former group than the latter group (Supplementary Table 3).

### Mortality and morbidity trends

**Fig. 3:**
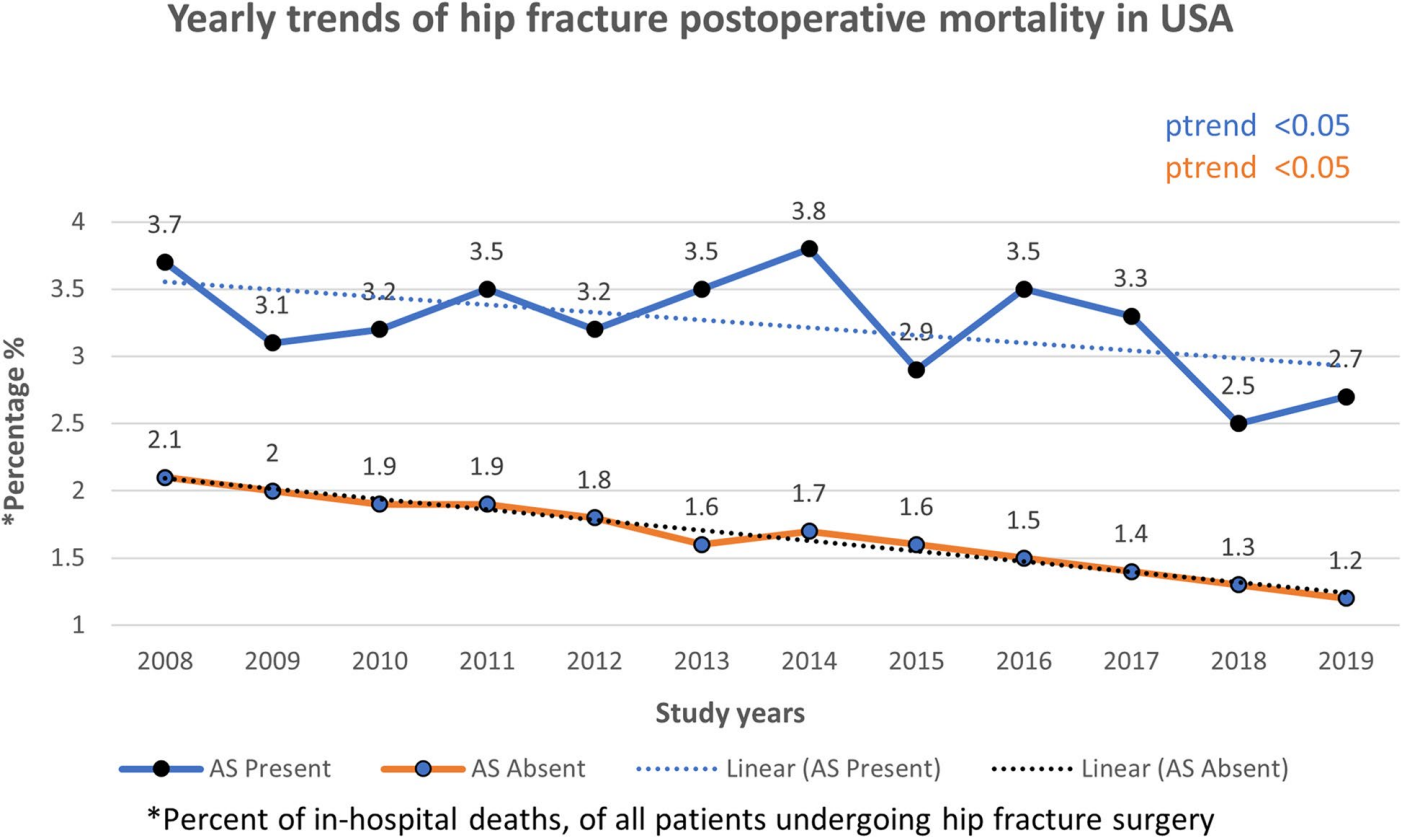
Yearly trends in hip fracture postoperative mortality

**Fig. 4:**
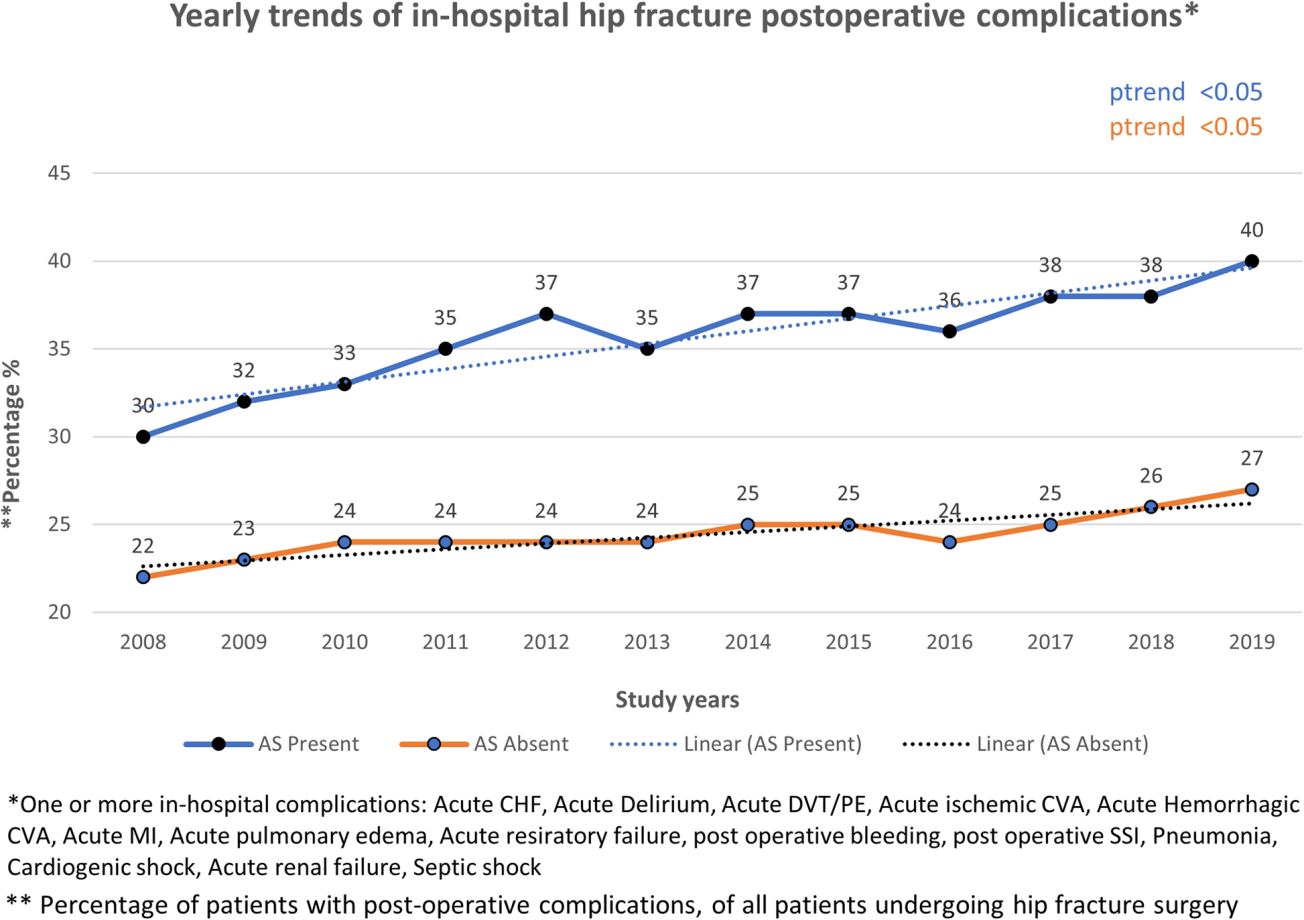
Yearly trends in hip fracture postoperative morbidity

In the study period of 2008 to 2019, there was a statistically significant (p-trend < 0.05) decline in hip fracture postoperative mortality in patients with and without AS (Fig. 3). There was an increase in the rate of one or more major postoperative complications in both groups (Fig. 4).

## Discussion

To our knowledge, this is the most comprehensive study of hospitalized hip fracture patients who underwent surgery with concomitant AS. We found that AS with hip fracture is common and was present in 3.3% of hospitalized patients. The in-hospital postoperative mortality rate of these patients was 3.3%. Notably, this rate decreased from 3.7% in 2008 to 2.7% in 2019 (p for trend < 0.05).

A significant proportion of patients (37.6%) experienced at least one major complication following hip fracture surgery. Importantly, AS was an independent predictor of postoperative in-hospital mortality. This association was particularly strong among AS patients with acutely decompensated CHF.

In population-based studies, some degree of AS is present in 1 in 10 patients aged over 75 years [21]. In our cohort of hospitalized patients with hip fracture, 1 in 30 had AS, and the prevalence was much higher in patients above 75 years of age. This is likely because our study is based on only hospitalized hip fracture patients. AS patients had higher burden of comorbidities in this study. CAD was present in 40% of hospitalized AS patients with hip fracture, which is in agreement with other studies where 44–50% of AS patients had underlying CAD [22, 23]. However, our study did not identify CAD as an independent predictor of postoperative mortality in AS patients. This finding might be attributed to the shared risk factors between AS and CAD. Patients are also routinely evaluated for CAD perioperatively due to its association with 30-day postoperative mortality [24] in both cardiac and noncardiac surgery, but evaluation of AS is not routinely performed perioperatively.

Progression of AS can lead to the development of decompensated CHF [25]. Within this study, 0.21% of all AS patients with hip fracture had acute or acute on chronic CHF. This subgroup experienced significantly higher postoperative in-hospital mortality and morbidity rates than AS patients without acute CHF. Patients may develop pulmonary hypertension (PH) with increasing severity of AS, a significant risk factor for postoperative mortality in noncardiac surgery. A study by Cignoni et al. [26] reported 2.5 times higher in-hospital mortality for patients undergoing hip fracture surgery with both AS and PH compared to patients with AS alone. In this study, PH in the adjusted analysis was independently associated with postoperative mortality (aOR 1.34, p < 0.001) in AS patients.

In one retrospective case–controlled review of elderly (≥ 65 years) hip fracture patients treated surgically, moderate/severe AS and pulmonary and renal diseases were identified as independent predictors of severe 30-day postoperative complications [16]. Our study corroborates these findings, with the strongest association of in-hospital mortality noted with age ≥ 85 years.

CKD and ESRD [27] are known risk factors for perioperative adverse cardiovascular events and postoperative mortality. They can predispose patients to changes in the sodium and fluid balance, vascular calcification, and inflammatory changes leading to atherosclerotic plaque destabilization [28]. In this analysis, both CKD (aOR 1.44, *p* < 0.001) and ESRD (aOR 3.40, *p* < 0.001) were associated with higher odds of postoperative mortality in AS patients. Malnutrition and poor functional status are associated with poor outcomes after hip fracture surgery. Malnourished patients are 7 times more likely to suffer complications and have a higher length of hospitalization than non-malnourished patients when admitted to the hospital with acetabular fracture and undergo surgery [29]. In our study, 5.7% of all patients admitted with hip fracture were malnourished, which was associated with higher odds of postoperative mortality (aOR 2.38, *p* < 0.001). The identification of malnutrition and benefits of nutritional interventions in hospitalized patients with hip fracture are not well studied.

Postoperative mortality in hip fracture patients with AS decreased in this study period from 2008–2019 (p-trend < 0.05). In the AHA/ACC guidelines on perioperative cardiovascular evaluation and management of patients undergoing noncardiac surgery [14], the use of 3 calculators [24, 30, 31] to assess a patient’s surgical risk is recommended. These risk calculators use different comorbidities as variables to calculate surgical risk; however, none of them take into consideration aortic valve function.

AS patients requiring noncardiac surgery pose a clinical challenge due to their higher mortality risk and likelihood of perioperative complications. This patient cohort could benefit from careful preoperative assessment, management of comorbid conditions, and surgical optimization through a multidisciplinary team. A careful preoperative assessment of volume status and invasive hemodynamic monitoring intraoperatively, especially in patients who have developed CHF and pulmonary hypertension from moderate to severe AS, can lead to improved outcomes.

According to the European Society of Cardiology preoperative cardiovascular disease management guidelines [32], asymptomatic patients with severe AS preparing for noncardiac surgery should be evaluated for potential aortic valve replacement, either surgical or transcatheter (Class IIa recommendation). Transcatheter aortic valve implantation (TAVI) in recent years has been identified as a minimally invasive option with improved outcomes in patients at higher surgical replacement risk [33]. However, utilization of TAVI and percutaneous balloon aortic valvuloplasty (BAV) in moderate to severe AS patients during the perioperative period for time-sensitive intermediate cardiac risk surgeries, such as hip fracture, remains understudied [34]. In this cohort, very few patients with hip fracture underwent TAVI in the same hospitalization. We were unable to determine if TAVI in high-risk patients before hip fracture surgery improves postoperative outcomes due to the absence of AS severity data in the NIS dataset. Given the increased postoperative mortality and morbidity rates associated with delays in hip fracture surgery [35–37], further research should investigate how deferring surgery for endovascular procedures such as TAVI or BAV may influence these outcomes. This highlights the importance of understanding the potential impacts of treatment timing on patient outcomes in this complex clinical scenario.

### Strengths and limitations of the study

In this retrospective cohort study on a large inpatient dataset, demographic features and comorbidities were taken into consideration while studying factors predicting outcomes such as in-hospital mortality and postoperative complications. We excluded patients with rheumatic and congenital AS from this analysis, as their presentation and course may differ from those of patients with calcific and age-related AS. The HCUP NIS database, however, does not specify the severity of AS by providing the valve area. To identify patients with clinically significant AS, we studied patients who had acutely decompensated CHF diagnosis while hospitalized for hip fracture and had underlying AS, which may over- or underestimate the actual severity of AS in this group of patients. Each patient in the NIS database represents an index hospitalization. This sample is not designed to follow patients longitudinally; hence, long-term outcomes such as mortality, complications after discharge and rate of readmissions cannot be studied. NIS identifies the diagnosis based on the ICD coding system, which may be subject to error; however, we identified the codes for conditions that are frequently used in inpatient settings. The timeline of events is not provided in the NIS dataset hence we are cautiously reporting association of variables with outcomes rather than causation.

## Conclusion

Patients with AS who undergo hip fracture surgery exhibit significantly higher in-hospital mortality and morbidity rates than non-AS patients. Although our study from 2008–2019 documented a decline in postoperative mortality among AS patients with hip fracture, the occurrence of in-hospital complications appears to be on the rise. A multidisciplinary approach is needed to identify high-risk patients, risk stratify and medically optimize them for surgery to reduce mortality and postoperative complications. Studies are required to discern whether deferring hip fracture surgery for procedures such as TAVI or percutaneous BAV in high-risk AS patients influences postoperative mortality and outcomes.

### Supplementary Information

**Additional file 1: Supplementary Table 1.** ICD 9 and ICD 10 codes. **Supplementary Table 2.** Baseline characteristics of hip fracture patients undergoing surgery. **Supplementary Table 3.** Outcomes post hip fracture surgery stratified by clinically significant AS (Acute CHF with AS).

**Additional file 2: Supplementry Table 4.** Checklist for working with the NIS.

**Additional file 3: Supplementary Table 5.** The RECORD statement – checklist of items, extended from the STROBE statement that should be reported in observational studies using routinely collected health data.

## Data Availability

The dataset analyzed during the current study is available at the HCUP website: https://hcup-us.ahrq.gov/

## Abbreviations

AS: Aortic stenosis
NIS: Nationwide inpatient sample
SD: Standard deviation
IQR: Interquartile range
ICD: International classification for disease
CHF: Congestive heart failure
AMI: Acute myocardial infarction
DVT: Deep venous thrombosis
PE: Pulmonary embolism
CVA: Cerebrovascular accident
COPD: Chronic obstructive pulmonary disease
SSI: Surgical site infection
CKD: Chronic kidney disease
ESRD: End-stage renal disease
CAD: Coronary artery disease
DM: Diabetes mellitus
OR: Odds ratio
aOR: Adjusted odds ratio
CI: Confidence interval
TAVI: Transcatheter aortic valve implantation
BAV: Balloon aortic valvuloplasty
