# Supplementary material for: Postoperative outcomes, predictors and trends of mortality and morbidity in patients undergoing hip fracture surgery with underlying aortic stenosis: a nationwide inpatient sample analysis
Source: BMC Cardiovasc Disord. 2023 Nov 3;23:535. doi: 10.1186/s12872-023-03584-2 (PMC10623838; doi:10.1186/s12872-023-03584-2)
Supplement: Supplementary file 1 — Additional file 1: Supplementary Table 1. ICD 9 and ICD 10 codes. Supplementary Table 2. Baseline characteristics of hip fracture patients undergoing surgery. Supplementary Table 3. Outcomes post hip fracture surgery stratified by clinically significant AS (Acute CHF with AS). [file 12872_2023_3584_MOESM1_ESM.docx]

**Supplementary Table 1. ICD 9 and ICD 10 codes**

|  | ICD – 9 codes | ICD – 10 codes |
| --- | --- | --- |
| Aortic Stenosis | 424.1 | I35.0 |
| Hip Fracture | 820.03, 820.13, 820.01,820.09, 820.8, 820.20, 820.30, 820.21, 820.00, 820.10, 820.31, 820.02, 820.12, 820.20, 820.8, 820.31, 820.9, 733.14, 733.15, 820.19, 733.96, 820.22, 820.30, 820.19, 820.22, 820.32, 820.01, 820.11, 820.9 | S72.001A, S72.001B, S72.001C, S72.002A, S72.002B, S72.002C, S72.009A, S72.009B, S72.009C, S72.011A, S72.011B, S72.011C, S72.012A, S72.012B, S72.012C, S72.019A, S72.019B. S72.019C, S72.021A, S72.021B, S72.021C, S72.022A, S72.022B, S72.022C,  S72.023A, S72.023B, S72.023C,  S72.024A, S72.024B, S72.024C,  S72.025A, S72.025B, S72.025C,  S72.026A, S72.026B, S72.026C,  S72.031A, S72.031B, S72.031C,  S72.032A, S72.032B, S72.032C,  S72.033A, S72.033B, S72.033C,  S72.034A, S72.034B, S72.034C,  S72.035A, S72.035B, S72.035C,  S72.036A, S72.036B, S72.036C,  S72.041A, S72.041B, S72.041C,  S72.042A, S72.042B, S72.042C,  S72.043A, S72.043B, S72.043C,  S72.044A, S72.044B, S72.044C,  S72.045A, S72.045B, S72.045C,  S72.046A, S72.046B, S72.046C,  S72.051A, S72.051B, S72.051C,  S72.052A, S72.052B, S72.052C,  S72.059A, S72.059B, S72.059C,  S72.061A, S72.061B, S72.061C,  S72.062A, S72.062B, S72.062C,  S72.063A, S72.063B, S72.063C,  S72.064A, S72.064B, S72.064C,  S72.065A, S72.065B, S72.065C,  S72.066A, S72.066B, S72.066C,  S72.091A, S72.091B, S72.091C,  S72.092A, S72.092B, S72.092C,  S72.099A, S72.099B, S72.099C,  S72.101A, S72.101B, S72.101C,  S72.102A, S72.102B, S72.102C,  S72.109A, S72.109B, S72.109C,  S72.111A, S72.111B, S72.111C,  S72.112A, S72.112B, S72.112C,  S72.113A, S72.113B, S72.113C,  S72.114A, S72.114B, S72.114C,  S72.115A, S72.115B, S72.115C,  S72.116A, S72.116B, S72.116C,  S72.121A, S72.121B, S72.121C,  S72.122A, S72.122B, S72.122C,  S72.123A, S72.123B, S72.123C,  S72.124A, S72.124B, S72.124C,  S72.125A, S72.125B, S72.125C,  S72.126A, S72.126B, S72.126C,  S72.131A, S72.131B, S72.131C,  S72.132A, S72.132B, S72.132C,  S72.133A, S72.133B, S72.133C,  S72.134A, S72.134B, S72.134C,  S72.135A, S72.135B, S72.135C,  S72.136A, S72.136B, S72.136C,  S72.141A, S72.141B, S72.141C,  S72.142A, S72.142B, S72.142C,  S72.143A, S72.143B, S72.143C,  S72.144A, S72.144B, S72.144C,  S72.145A, S72.145B, S72.145C,  S72.146A, S72.146B, S72.146C,  S72.21XA, S72.21XB, S72.21XC,  S72.22XA, S72.22XB, S72.22XC,  S72.23XA, S72.23XB, S72.23XC,  S72.24XA, S72.24XB, S72.24XC,  S72.25XA, S72.25XB, S72.25XC,  S72.26XA, S72.26XB, S72.26XC, |
| Hip Fracture surgery | 79.15, 79.25, 79.35, 79.55, 81.21, 81.40, 81.51, 81.52, 81.53 | 0SRB019, 0SRB01A, 0SRB01Z, 0SRB01Z, 0SRB029, 0SRB02A, 0SRB02Z, 0SRB039, 0SRB03A, 0SRB03Z, 0SRB049, 0SRB04A, 0SRB04Z, 0SRB069, 0SRB06A, 0SRB06Z, 0SRB0J9, 0SRB0JA, 0SRB0JZ, 0SR9019, 0SR901A, 0SR901Z, 0SR9029, 0SR902A, 0SR902Z, 0SR9039, 0SR903A, 0SR903Z, 0SR9049, 0SR904A, 0SR904Z, 0SR9069, 0SR906A, 0SR906Z, 0SR90EZ, 0SR90J9, 0SR90JA, 0SR90JZ, 0SR90KZ, 0QS604Z, 0QS605Z, 0QS606Z, 0QS60BZ, 0QS60CZ, 0QS60DZ, 0QS60ZZ, 0QS634Z, 0QS60635Z, 0QS636Z, 0QS63BZ, 0QS63CZ, 0QS63DZ, 0QS63ZZ, 0QS644Z, 0QS645Z, 0QS646Z, 0QS64BZ, 0QS64CZ, 0QS64ZZ, 0QS6XZZ, 0QS704Z, 0QS705Z, 0QS706Z, 0QS70BZ, 0QS70CZ, 0QS70DZ, 0QS70ZZ, 0QS734Z, 0QS735Z, 0QS736Z, 0QS73BZ, 0QS73CZ, 0QS73DZ, 0QS73ZZ, 0QS744Z, 0QS745Z, 0QS746Z, 0QS74BZ, 0QS74CZ, 0QS74DZ, 0QS74ZZ, 0QS7XZZ, 0SR9019, 0SR901A, 0SR901Z, 0SR9029, 0SR902A, 0SR902Z, 0SR9039, 0SR903A, 0SR903Z, 0SR9049, 0SR904A, 0SR904Z, 0SR9069, 0SR906A, 0SR906Z, 0SR90J9, 0SR90JA, 0SR90JZ, 0SRB019, 0SRB01A, 0SRB01Z, 0SRB029, 0SRB02A, 0SRB02Z, 0SRB039, 0SRB03A, 0SRB03Z, 0SRB049, 0SRB04A, 0SRB04Z, 0SRB069, 0SRB06A, 0SRB06Z, 0SRB0J9, 0SRB0JA, 0SRB0JZ, 0SRR019, 0SRR01A, 0SRR01Z, 0SRR039, 0SRR03A, 0SRR03Z, 0SRR0J9, 0SRR0JA, 0SRR0JZ, 0SRS019, 0SRS01A, 0SRS01Z, 0SRS039, 0SRS03A, 0SRS03Z, 0SRS0J9, 0SRS0JA, 0SRS0JZ, 0QR60JZ, 0QR63JZ, 0QR64JZ, 0QR70JZ, 0QR73JZ, 0QR74JZ, 0QQ60ZZ, 0QQ63ZZ, 0QQ64ZZ, 0QQ70ZZ, 0QQ73ZZ, 0QQ74ZZ, 0QS604Z, 0QS606Z, 0QS634Z, 0QS636Z, 0QS644Z, 0QS646Z, 0QS704Z, 0QS706Z, 0QS734Z, 0QS736Z, 0QS744Z, 0QS746Z, 0QH604Z, 0QH606Z, 0QH634Z, 0QH636Z, 0QH644Z, 0QH646Z, 0QH704Z, 0QH706Z, 0QH734Z, 0QH736Z, 0QH744Z, 0QH746Z, 0SQ90ZZ, 0SQ93ZZ, 0SQ94ZZ, 0SQB0ZZ |
| Acute Myocardial Infarction | 410 | I21 |
| Post operative surgical site infection | 998.59 | T81.4 |
| Cardiogenic Shock | 785.51 | R57.0 |
| Septic Shock | 785.52 | R65.21 |
| Acute Respiratory Failure | 518.81, 518.51 | J96 |
| Acute pulmonary edema | 518.4, 542.81 | J81.0 |
| Acute Heart failure decompensation | 428.21., 428.23, 428.31, 428.33, 428.41, 428.43 | I50.21, I50.23, I50.31, I50.33, I50.41, I50.43 |
| Acute DVT/PE | 415.1, 453.8, 453.4 | I82.40, I26.99 |
| Pneumonia | 481, 482, 483, 485, 486, 507.0, 997.31 | J18, J15, J69 |
| Acute renal failure | 584 | N17 |
| Acute Delirium | 2930 | F05 |
| Post-op bleeding | 998.1 | L76.22 |
| Acute ischemic CVA | 433, 434, 436, 997 | I63 |
| Acute hemorrhagic CVA | 430, 431, 432 | I60, I61, I62 |
| Pulmonary hypertension | 416 | I27 |
| Ataxia | 438.84, 3343, 781.2, 781.3 | R26, R27, A52.11, G119, G32.81 |
| Malnutrition | 262, 263, 261 | E43, E44 |
| Obesity | 278.00, 278.01 | E66.9, E66.8, E66.1, E66.01, E66.09 |
| Dementia | 294.20, 294.21, 290.9, 295.9, 294.11, 04611, 331.82, 331.19, 292.82, 290.40 | F01, F02, F03, G10, G30, G31, G32 |
| Vitamin D deficiency | 268 | E55 |
| Peripheral vascular disease | 440, 443, 445 | I70, I73, I74, I75 |
| Congestive heart failure | 428.22, 428.32, 428.42, 402.01, 402.11, 402.91 | I42, I43, I50.22, I50.32, I50.42, I50.812, I50.82, I50.83, I50.84, I50.89. I50.9 |
| Alcohol abuse | 303, 305.0 | F10.1 |
| Coronary artery disease | 412, 413, 414, 440.0 | I20, I25, Z98.61 |
| Long term use of anticoagulation | V58.61 | Z79.01 |
| End stage renal disease | 585.6 | N186 |
| Chronic kidney disease | 585.9, 585.1, 585.2, 585.3, 585.4, 585.5 | N18.1, N18.2, N18.3, N18.4, N18.5, N18.9 |
| Osteoporosis | 733.0 | M81.0, M81.6, M81.8 |
| Smoking | 305.1 | F17.219, Z72.0 |
| Osteoarthritis | 715, 721, 719, 724 | M15, M16, M17, M18, M19 |
| Rheumatoid arthritis | 714 | M05, M06 |

| Supplementary Table 2. Baseline characteristics of hip fracture patients undergoing surgery | | | | |
| --- | --- | --- | --- | --- |
|  | | **Aortic stenosis** | | **p value** |
|  | Total, No. (%) | **Present** | **Absent** |  |
|  | 2,834,919 | 94,270 (3.3) | 2,740,649 (96.7) |  |
| Characteristics | | | | |
| Age (years) <0.001 | | | | |
| Mean + SD |  | 84.5 + 6.8 | 77.1 + 13.0 |  |
| Age (years) |  |  |  | <0.001 |
| 18-54 | 130,060 (5.4) | 205 (0.2) | 129,855 (5.6) |  |
| 55 - 64 | 216,900 (9.0) | 1,195 (1.5) | 215,705 (9.3) |  |
| 65 - 74 | 417,790 (17.4) | 5,790 (7.2) | 412,000 (17.8) |  |
| 75 - 84 | 736,700 (30.7) | 22,835 (28.4) | 713,865 (30.8) |  |
| >85 | 897,265 (37.4) | 50,280 (62.6) | 846,985 (36.5) |  |
| Gender |  |  |  | 0.83 |
| Female | 1,634,495 (68.1) | 54,775 (68.2) | 1,579,720 (68.1) |  |
| Male | 764,005 (32.0) | 25,515 (31.8) | 738,490 (32.0) |  |
| Race |  |  |  | <0.001 |
| White | 1,975,275 (85.9) | 68,480 (88.7) | 1,906,795 (85.8) |  |
| Black | 102,955 (4.4) | 2,060 (2.6) | 122,220 (5.5) |  |
| Hispanics | 125,845 (5.4) | 3,625 (4.7) | 122,220 (5.5) |  |
| Other | 96,375 (4.2) | 3,030 (3.9) | 93,345 (4.2) |  |
| Comorbidities | | | | |
| Hypertension | 490,365 (20.4) | 26,715 (33.4) | 463,650 (20.0) | <0.001 |
| Diabetes | 542,455 (22.6) | 19,770 (24.6) | 522,685 (22.5) | <0.001 |
| Pulmonary Hypertension | 107,180 (4.4) | 10,705 (13.3) | 96,475 (4.1) | <0.001 |
| Coronary artery disease | 581,930 (24.3) | 32,150 (40.0) | 59,780 (23.7) | <0.001 |
| Peripheral vascular disease | 188,340 (7.8) | 10,315 (12.8) | 178,025 (7.6) | <0.001 |
| Congestive heart failure | 393,820 (16.4) | 27,920 (34.8) | 365,900 (15.8) | <0.001 |
| Cardiac Arrhythmias | 715,970 (29.8) | 37,655 (47.0) | 678,315 (29.3) | <0.001 |
| Long term anticoagulation | 232,525 (9.6) | 10,395 (13.0) | 222,130 (9.5) | <0.001 |
| Chronic kidney disease | 390,100 (16.3) | 20,065 (25) | 370,035(16) | <0.001 |
| End stage renal disease | 44,745 (1.8) | 2,005 (2.5) | 42,740 (1.8) | <0.001 |
| Malignancy | 26,400(3.0) | 845(3.2) | 25,555 (3.0) | 0.36 |
| Obesity | 123,900 (5.1) | 3,885 (4.8) | 120,015 (5.1) | 0.06 |
| Alcohol Abuse | 109,185 (4.5) | 1,560 (1.9) | 107,625 (4.6) | <0.001 |
| Ataxia | 55,845 (2.3) | 2,205 (2.7) | 53,640 (2.3) | <0.001 |
| Osteoporosis | 419,995 (17.5) | 15,715 (19.6) | 404,280 (17.4) | <0.001 |
| Vitamin D deficiency | 94,565 (3.9) | 3,095 (3.8) | 91,470 (3.9) | 0.55 |
| Protein energy malnutrition | 130,805 (5.4) | 4,685 (5.8) | 126,120 (5.4) | 0.032 |
| Dementia | 523,285 (21.8) | 21,705 (27) | 501,580 (21.6) | <0.001 |
| Osteoarthritis | 447,935 (18.7) | 17,385 (21.6) | 430,550 (18.6) | <0.001 |
| Rheumatoid arthritis | 64,110 (2.6) | 2,160 (2.7) | 61,950 (2.6) | 0.90 |
| Smoking | 140,930 (5.8) | 2,555 (3.1) | 138,375 (5.9) | <0.001 |
| Chronic obstructive pulmonary disease | 193,120 (22.0) | 5,860 (22.3) | 187,260 (22.0) | 0.54 |
| Elixhauser groups |  | | | <0.001 |
| <4 | 1,860,890 (77.6) | 51,950 (64.7) | 1,808,940 (78) |  |
| 4 - 6 | 440,505 (18.4) | 19,885 (24.8) | 420,620 (18.1) |  |
| >6 | 97,320 (4.06) | 8,470 (10.5) | 88,850 (3.8) |  |
| Insurance status | | | | <0.001 |
| Medicare | 1,966,495 (82) | 74,350 (92.6) | 1,892,145 (81.6) |  |
| Medicaid | 85,770 (3.5) | 790 (0.9) | 84,980 (3.6) |  |
| Private | 249,110 (10.4) | 3,930 (4.9) | 245,180 (10.6) |  |
| Others | 97,340 (4.0) | 1,235 (1.5) | 96,105 (4.1) |  |
| Hospital Bed size | | | | 0.23 |
| Small | 447,910 (18.7) | 14,640 (18.2) | 433,270 (18.7) |  |
| Medium | 724,455 (30.2) | 24,090 (30) | 700,365 (30.2) |  |
| Large | 1,226,350 (51.1) | 41,575 (51.8) | 1,184,775 (51.1) |  |
| Hospital Location | | | | <0.001 |
| Rural | 289,205 (12.1) | 8,030 (10) | 281,175 (12.1) |  |
| Urban | 2,109,510 (87.9) | 72,275 (90) | 2,037,235 (87.9) |  |
|  |  |  |  |  |

| Supplementary Table 3. Outcomes post hip fracture surgery stratified by clinically significant AS (Acute CHF with AS) | | | | |
| --- | --- | --- | --- | --- |
| Outcomes | No. (%) | **Clinically significant Aortic Stenosis** | | **p Value** |
|  |  | **Present** | **Absent** |  |
|  | 2,398,714 (84.6) | 5,135 (0.21) | 2,392,324 (99.8) |  |
| Length of stay - days  Median (IQR) |  | 8(5-11) | 5(4-7) | <0.001 |
| In hospital Deaths | 38,995 (1.6) | 485 (9.4) | 38,510 (1.6) | <0.001 |
| Acute Myocardial Infarction | 39,495 (1.6) | 695 (13.5) | 38,800(1.6) | <0.001 |
| Acute Pulmonary edema | 3,980 (0.1) | 40(0.7) | 3,940 (0.1) | <0.001 |
| Cardiogenic shock | 4,330 (0.18) | 195 (3.8) | 4,135 (0.1) | <0.001 |
| Acute DVT/Pulmonary embolism | 25,890 (1.1) | 100 (1.9) | 25,790 (1.1) | 0.006 |
| Septic shock | 10,155 (0.42) | 95 (1.8) | 10,060 (0.4) | <0.001 |
| Pneumonia | 137,290 (5.7) | 1,150 (22.4) | 136,140 (5.69) | <0.001 |
| Acute Respiratory failure | 146,620 (6.1) | 1,750 (34) | 144,870 (6.0) | <0.001 |
| Acute Ischemic CVA | 66,960 (2.79) | 385 (7.5) | 66,575 (2.7) | <0.001 |
| Acute Renal failure | 306,800 (12.8) | 1,970 (38.3) | 304,830 (12.7) | <0.001 |
| Acute Delirium | 64,065 (2.6) | 285 (5.5) | 63,780 (2.6) | <0.001 |
| Post operative SS infection | 4,135 (0.1) | 35(0.6) | 4,100 (0.1) | <0.001 |
| Post operative bleeding | 14,495 (0.60) | 60(1.1) | 14,435 (0.6) | 0.019 |
| Abbreviations:  IQR: Interquartile range  DVT: Deep venous thrombosis  CVA: Cerebrovascular accident  SS: Surgical site |  |  |  |  |
