# Supplementary material for: Postoperative outcomes, predictors and trends of mortality and morbidity in patients undergoing hip fracture surgery with underlying aortic stenosis: a nationwide inpatient sample analysis
Source: BMC Cardiovasc Disord. 2023 Nov 3;23:535. doi: 10.1186/s12872-023-03584-2 (PMC10623838; doi:10.1186/s12872-023-03584-2)
Supplement: Supplementary file 2 — Additional file 2: Supplementry Table 4. Checklist for working with the NIS. [file 12872_2023_3584_MOESM2_ESM.docx]

**Supplementry Table 4. CHECKLIST FOR WORKING WITH THE NIS**

|  | **Checklist Item** | **Description** | **Checklist Resource** |
| --- | --- | --- | --- |
| ☒ | Obtain and adhere to the HCUP Nationwide Database Data Use Agreement (DUA).^a^ | The HCUP DUA governs the disclosure and use of the data, including affirmations to protect individuals, establishments, and the database itself. | For general information, review the [*Responsibilities of the Data Purchaser*](https://hcup-us.ahrq.gov/tech_assist/centdist/ResponsibilitiesDataPurchaser.pdf) and the [*HCUP Nationwide Database Data Use Agreement (DUA)*](https://hcup-us.ahrq.gov/team/NationwideDUA.pdf).  To access the NIS, you must complete the [HCUP Data Use Agreement Training](https://hcup-us.ahrq.gov/tech_assist/dua.jsp). |
| ☒ | Verify privacy protections for individuals and hospitals. | Individuals cannot be identified directly or indirectly.  Reporting cell sizes < 10 increases the risk of re-identification and is discouraged, as specified in the Data Use Agreement.  At least two hospitals must contribute to each cell. | For general information, review the [*Requirements for Publishing with HCUP Data*](https://hcup-us.ahrq.gov/db/publishing.jsp) page on the HCUP User Support (HCUP-US) website. |
| ☒ | Cite HCUP, the NIS, and other HCUP tools. | HCUP, the NIS, and other supporting tools must be correctly cited in the abstract and manuscript. | For more information, review the [*Suggested Citations for HCUP Databases and Tools*](https://hcup-us.ahrq.gov/tech_assist/citations.jsp) page on HCUP-US. |
| N/A | Acknowledge HCUP Partners. | Participating HCUP Partners should be listed in the manuscript by name or acknowledged by a hyperlink to the HCUP-US website. | For more information, review the [*List of HCUP Data Partners for Reference in Publications*](https://hcup-us.ahrq.gov/db/hcupdatapartners.jsp) page on HCUP-US. |
| **Research Design** | | | |
| ☒ | Learn how to account for the NIS sampling design. | The NIS is sampled from the HCUP State Inpatient Databases (SID). Accounting for the sampling design is critical for accurate analyses. | For detailed information, review the [*HCUP Methods Report# 2014-04: Nationwide Inpatient Sample (NIS) Redesign Report*](https://hcup-us.ahrq.gov/reports/methods/2014-04.pdf).  To learn more about the NIS sample design, view the *Sample Design On-line Tutorial* on the [*Tutorial Series*](https://hcup-us.ahrq.gov/tech_assist/tutorials.jsp) page. |
| ☒ | Only inpatient events are captured in the NIS. | The unit of analysis in the NIS is inpatient stays, not individual patients. Only conditions, procedures, and diagnostic tests occurring during a specific inpatient hospital encounter are captured in the NIS. Records of events and diagnoses before or after the stay are not available. | For more information, review the *Contents of the NIS* section of the *Introduction to the NIS* on the [*NIS Database Documentation*](https://hcup-us.ahrq.gov/db/nation/nis/nisdbdocumentation.jsp) page.  For more information on conducting revisit analyses at the national level, review the [*Nationwide Readmissions Database (NRD)*](https://hcup-us.ahrq.gov/nrdoverview.jsp). For State-level information, review the [*HCUP Supplemental Variables for Revisit Analyses*](https://hcup-us.ahrq.gov/toolssoftware/revisit/revisit.jsp). |
| ☒ | Excluded Facilities | The NIS includes community hospitals, but it excludes rehabilitation or long-term acute care (LTAC) hospitals. | Additional information on hospital-level exclusions is included in the *Introduction to the NIS* on the [*NIS Database Documentation page*](https://hcup-us.ahrq.gov/db/nation/nis/nisdbdocumentation.jsp). |
| ☒ | No State-level analyses are performed. | The sampling design of the NIS does not support State-level analyses. The SID must be used for State-level research. | For more information, review [*Why the NIS Should Not Be Used to Make State-Level Estimates*](https://hcup-us.ahrq.gov/db/nation/nis/nis_statelevelestimates.jsp).  To learn more about the SID, review the [*Overview of the State Inpatient Databases (SID)*](https://hcup-us.ahrq.gov/sidoverview.jsp) page on HCUP-US. |
|  |  |  |  |
| ☒ | Facility-level analyses are limited. | Starting with 2012, the sampling design of the NIS does not support hospital-level totals because only a sample of discharges from each hospital in the sampling frame are included in the NIS, and hospital sampling rates vary. However, hospital percentages (e.g. percent Medicare patients) can be estimated.  Prior to 2012, the NIS was a sample of U.S. community hospitals and could support studies with hospitals as the unit of analysis because all discharges from each sampled hospital were included in the NIS, although different hospitals were sampled each year.  Users should not attempt to identify individual facilities as specified in the Data Use Agreement. | For more information, review the "Sampling Design of the NIS" section of the *Introduction to the NIS* on the [*NIS Database Documentation*](https://hcup-us.ahrq.gov/db/nation/nis/nisdbdocumentation.jsp) page on HCUP-US. |
| ☒ | No physician-level analyses are performed. | The NIS does not include physician identifiers. | For more information, review the [*NIS Description of Data Elements*](https://hcup-us.ahrq.gov/db/nation/nis/nisdde.jsp) page on HCUP-US. |
|  |  |  |  |
| ☒ | It is not possible to track patients in the NIS. | The NIS does not include patient identifiers. | For more information, review the *Introduction to the NIS* on the [*NIS Database Documentation*](https://hcup-us.ahrq.gov/db/nation/nis/nisdbdocumentation.jsp) page on HCUP-US. |
| ☒ | Administrative (ICD) codes are appropriate for the outcomes of interest. | Administrative codes for the conditions or procedures of interest (ICD-9-CM and ICD-10-CM/PCS,) should be selected with care, especially over time, as codes and coding rules change annually. | For more information, review the *Choosing Data Elements for Analysis* section of the *Introduction to the NIS* on the [*NIS Database Documentation*](https://hcup-us.ahrq.gov/db/nation/nis/nisdbdocumentation.jsp) page on HCUP-US.  Refer to the [ICD-10-CM/PCS Resources](https://hcup-us.ahrq.gov/datainnovations/icd10_resources.jsp) page on HCUP-US under Data Innovations for a summary of key issues for researchers using HCUP and other administrative databases that include ICD-10-CM/PCS coding.  To check for year-to-year variation in administrative codes, consult with a medical coding professional. |
|  |  |  |  |
| ☒ | Comorbidities must be distinguished from complications. | Secondary diagnosis codes in the NIS do not differentiate comorbidities from complications, unless they are specific to in-hospital events captured by a specific ICD code that indicates a complication.  Select comorbidities are identified by the Elixhauser Comorbidity Software for ICD-9-CM or Elixhauser Comorbidity Software Refined for ICD-10-CM. Data elements derived from these tools are included on the NIS Severity File through quarter 3 of data year 2015 and the NIS Diagnosis and Procedure Groups File beginning data year 2019. | For more information, review the HCUP Methods Series Report # 2004-01, [*Comorbidity Software Documentation*](https://hcup-us.ahrq.gov/reports/methods/ComorbiditySoftwareDocumentationFinal.pdf) and the *Elixhauser Comorbidity Software for ICD-9-CM* or *Elixhauser Comorbidity Software Refined for ICD-10-CM* pages on the HCUP-US website. |
|  |  |  |  |
| ☒ | Account for year- based differences in data element availability in the NIS. | The study design should account for differences in data element availability across data years. For example, the number of diagnosis codes present can vary by year. | For more information about data element availability in the NIS, review the [*NIS Description of Data Elements*](https://hcup-us.ahrq.gov/db/nation/nis/nisdde.jsp) page on HCUP-US. |
| **Data Analysis** | | | |
| ☒ | Use weights for national estimates. | To generate national estimates using the NIS, use the discharge-level weight (DISCWT) to estimate discharges treated at community hospitals (excluding rehabilitation and LTAC facilities) in the United States.  To generate national estimates using multiple years of the NIS, you must apply weights using the variable TRENDWT (for data years prior to 2012) and the variable DISCWT (for data years 2012 and later). | For general information on weights, review [*Trend Weights for HCUP NIS Data*](https://hcup-us.ahrq.gov/db/nation/nis/trendwghts.jsp).  To learn how to apply NIS weights, view the [*Producing National HCUP Estimates On-line Tutorial*](https://hcup-us.ahrq.gov/tech_assist/tutorials.jsp) and review [*HCUP Methods Series Report# 2006-05: Using the HCUP National Inpatient Sample to Estimate Trends (Revised 12/15/15).*](https://hcup-us.ahrq.gov/reports/methods/2006_05_NISTrendsReport_1988-2004.pdf)  To learn how to apply the trend weights for multi-year analyses, view the *HCUP Multi-Year Analysis On-line Tutorial* on the [*Tutorial Series*](https://hcup-us.ahrq.gov/tech_assist/tutorials.jsp) page. |
| ☒ | Account for the design of the NIS when calculating standard errors. | Standard error calculations should take into account the stratification (data element NIS_STRATUM) and hospitals defining the clusters (data element HOSP_NIS). | For information applicable to data years 2012 and later, review [*HCUP Methods Series Report# 2015-09: Calculating National Inpatient Sample (NIS) Variances for Data Years 2012 and Later*](https://www.hcup-us.ahrq.gov/reports/methods/2015-09.pdf).  For information applicable to data years 2011 and earlier, review [*HCUP Methods Series Report# 2003-02: Calculating National Nationwide Inpatient Sample (NIS) Variances for Data Years 2011 and Earlier*](https://www.hcup-us.ahrq.gov/reports/methods/2003_02.pdf).  To learn how to calculate standard errors, view the *HCUP Calculating Standard Errors On-line Tutorial* on the [*Tutorial Series*](https://www.hcup-us.ahrq.gov/tech_assist/tutorials.jsp) page. |
|  |  |  |  |
| N/A | Account for clustering or nesting of observations. | Discharges in the NIS are clustered, or nested, within hospitals. Hierarchical linear modeling (HLM) is one way to account for this design aspect of the NIS. | For information on using HLM with the NIS, review the [*HCUP Methods Series Report# 2007-01: Hierarchical Modeling Using HCUP Data*](https://www.hcup-us.ahrq.gov/reports/methods/2007_01.pdf). |
|  |  |  |  |
| ☒ | Account for missing values. | Several techniques are available to assess and reduce the impact of missing data when using the NIS. | For general information, review the *Missing Values* section of the *Introduction to the NIS* on the [*NIS Database Documentation*](https://www.hcup-us.ahrq.gov/db/nation/nis/nisdbdocumentation.jsp) page.  For detailed information, review the [*HCUP Methods Report# 2015-01: Missing Data Methods for the NIS and SID.*](https://www.hcup-us.ahrq.gov/reports/methods/2015_01.pdf) |
|  |  |  |  |
| ☒ | Calculate rates of hospital care events per population when you need to control for differences in the underlying populations. | There are several sources of population data that can be used with the HCUP databases to calculate rates of hospital care events per population to improve comparisons between subgroups (e.g., region of the country). | More information is available under *Population Denominator Data for Use with the HCUP Databases* (multiple documents; updated annually) on the [*HCUP Methods Series Reports by Topic*](https://hcup-us.ahrq.gov/reports/methods/methods_topic.jsp#pop) page on HCUP-US. |
|  |  |  |  |
| ☒ | Estimate incidence or prevalence. | The NIS can be used to estimate incidence or prevalence of both common and rare conditions in some, but not all scenarios. | For information on estimating incidence and prevalence, review the [*HCUP Methods Series Report# 2016-06: Using the HCUP Databases to Study Incidence and Prevalence.*](https://www.hcup-us.ahrq.gov/reports/methods/2016-06.pdf) |
|  | | | |
| **ICD-9-CM to ICD-10-CM/PCS Transition** | | | |
| ☒ | Account for changes in the NIS related to ICD-10-CM/PCS. | The transition to ICD-10-CM/PCS has had a direct impact on the reporting of medical services, and these changes affect research using administrative data.  The structure of and data elements included in the NIS are affected by the transition to ICD-10-CM/PCS. | For more information, refer to the [*ICD-10-CM/PCS Resources*](https://hcup-us.ahrq.gov/datainnovations/icd10_resources.jsp) page on HCUP-US that summarizes key issues for researchers using HCUP and other administrative databases that include ICD-9-CM and ICD-10-CM/PCS coding.  For additional information about these changes, review the *2015 NIS Revised File Structure and New Data Elements* and *NIS Changes Beginning Data Year 2016* documents on the [*NIS Database Documentation*](https://hcup-us.ahrq.gov/db/nation/nis/nisdbdocumentation.jsp) page on HCUP-US. |
|  |  |  |  |
| ☒ | Follow HCUP recommendations for reporting trends with data that include both ICD-9-CM and ICD-10-CM/PCS coding. | Recommendations for reporting trends based on HCUP data that span the October 1, 2015 transition date (before and after the introduction of ICD-10-CM/PCS) have been developed to help researchers design studies. | For more information, review the [*Recommendations for Reporting Trends Using ICD-9-CM and ICD-10-CM/PCS Data*](https://www.hcup-us.ahrq.gov/datainnovations/HCUP_RecomForReportingTrends_070517.pdf). |
|  |  |  |  |
| ☒ | Use current versions of HCUP Tools for ICD-10-CM/PCS-coded data. | ICD-10-CM/PCS coding guidance is continuing to evolve. HCUP software tools for ICD-10-CM/PCS will be updated and should be reapplied throughout the research process. For this reason, it is important to always use the most current version of these tools. | Consult the [HCUP Tools & Software](https://hcup-us.ahrq.gov/tools_software.jsp) page on HCUP-US regularly for the most current versions of the HCUP software tools. |
